# Fenofibrate to prevent amputation and reduce vascular complications in patients with diabetes: FENO-PREVENT

**DOI:** 10.1186/s12933-024-02422-9

**Published:** 2024-09-03

**Authors:** Eu Jeong Ku, Bongseong Kim, Kyungdo Han, Seung-Hwan Lee, Hyuk-Sang Kwon

**Affiliations:** 1 Department of Internal Medicine, Seoul National University Hospital Healthcare System Gangnam Center, Seoul, South Korea; 2 Department of Internal Medicine, Seoul National University College of Medicine, Seoul, South Korea; 3 Department of Statistics and Actuarial Science, Soongsil University, Seoul, South Korea; 4 Division of Endocrinology and Metabolism, Department of Internal Medicine, Seoul St. Mary’s Hospital, College of Medicine, The Catholic University of Korea, Seoul, South Korea; 5 Department of Medical Informatics, College of Medicine, The Catholic University of Korea, Seoul, South Korea; 6 Division of Endocrinology and Metabolism, Department of Internal Medicine, Yeouido St. Mary’s Hospital, The Catholic University of Korea, Seoul, South Korea

**Keywords:** Amputation, Fenofibrate, Peripheral arterial disease, Type 2 diabetes mellitus

## Abstract

**Background:**

The potential preventive effect of fenofibrate on lower extremity amputation (LEA) and peripheral arterial disease (PAD) in patients with type 2 diabetes (T2D) is not fully elucidated.

**Methods:**

We selected adult patients ≥ 20 years of age with T2D from the Korean National Health Insurance Service Database (2009–2012). The fenofibrate users were matched in a 1:4 ratio with non-users using propensity scores (PS). The outcome variables were a composite of LEA and PAD and the individual components. The risks of outcomes were implemented as hazard ratio (HR) with 95% confidence intervals (CI). For safety issues, the risks of acute kidney injury, rhabdomyolysis and resulting hospitalization were analyzed.

**Results:**

A total of 114,920 patients was included in the analysis with a median follow-up duration of 7.6 years (22,984 and 91,936 patients for the fenofibrate user and non-user groups, respectively). After PS matching, both groups were well balanced. The fenofibrate group was associated with significantly lower risks of composite outcome of LEA and PAD (HR 0.81; 95% CI 0.70–0.94), LEA (HR 0.76; 95% CI 0.60–0.96), and PAD (HR 0.81; 95% CI 0.68–0.96). The risk of acute kidney injury, rhabdomyolysis, or hospitalization for these events showed no significant difference between the two groups. Subgroup analyses revealed consistent benefits across age groups, genders, and baseline lipid profiles.

**Conclusions:**

This nationwide population-based retrospective observational study suggests that fenofibrate can prevent LEA and PAD in patients with T2D who are on statin therapy.

**Supplementary Information:**

The online version contains supplementary material available at 10.1186/s12933-024-02422-9.

## Introduction

Type 2 diabetes (T2D) represents a significant public health concern, characterized by a rapidly increasing global prevalence [1]. Among the severe complications associated with T2D, the heightened risk of peripheral arterial disease (PAD) and lower extremity amputation (LEA) is of particular concern. PAD is an atherosclerotic occlusive disease of the lower extremities, constituting a major form of cardiovascular disease that encompasses both coronary heart disease and cerebrovascular disease [2, 3]. The prognosis for patients undergoing amputation due to diabetes is poor, with a mortality rate of 48% within the first-year post-amputation, increasing to > 70% within five years [4]. In a study in the United States, patients requiring amputations incur 3–6-fold longer hospital stays and significantly higher treatment costs than individuals without such interventions [3]. These conditions severely diminish quality of life and pose a major socioeconomic challenge, reflecting the substantial costs associated with long-term care, rehabilitation, and overarching burden on healthcare systems [5–8].

Recent clinical guidelines underscore the critical importance of comprehensive management strategies, including lipid-lowering therapy, smoking cessation, intensive glycemic control, and blood pressure management, to mitigate the risk of PAD in individuals with diabetes [9–11]. Statins, the cornerstone of lipid-lowering therapy, have markedly reduced cardiovascular risk but leave a substantial residual risk, even with well-controlled low-density lipoprotein cholesterol (LDL-C) levels [12–14]. This recognition has spotlighted triglycerides as a crucial therapeutic target for addressing residual cardiovascular risk, although pharmacotherapy aimed at triglycerides has produced mixed outcomes.

The prevalent dyslipidemia pattern in patients with diabetes is elevated triglycerides, reduced high-density lipoprotein cholesterol (HDL-C), and increased small dense LDL-C [15]. Despite the acknowledged necessity to manage atherogenic dyslipidemia in T2D patients for reducing residual cardiovascular risk, the effectiveness of fenofibrate, a medication targeted at reducing triglyceride levels, remains contentious [9, 16–18]. Notably, the Fenofibrate Intervention and Endpoint Lowering in Diabetes (FIELD) study and the Action to Control Cardiovascular Risk in Diabetes (ACCORD)-Lipid trial did not conclusively demonstrate a protective effect of fenofibrate against major cardiovascular events in the broad diabetic population [19, 20]. However, subgroup analyses have indicated that specific subgroups with T2D, particularly patients with significant hypertriglyceridemia and low HDL-C, might benefit from fenofibrate therapy, indicating the potential for personalized treatment approaches.

Due to the mixed evidence regarding the effectiveness of fenofibrate and the critical need for effective strategies to reduce the incidence of PAD and LEA among patients with T2D, this study aimed to investigate the real-world effects of fenofibrate on these outcomes.

## Methods

### Study design and data sources

The Korean National Health Insurance Service (NHIS) database was used in this retrospective observational cohort study. In South Korea, the NHIS is mandatory for the entire population of approximately 50 million people and provides comprehensive medical coverage to Korean nationals. The database contains demographic information such as age, sex, area of residence, and all healthcare utilization data including diagnoses, procedures, medication prescriptions, and surgical codes. Details on the database profile are described elsewhere [21]. The Institutional Review Board (IRB) of Seoul National University Hospital exempted informed consent because the database was provided to the researchers after anonymization (IRB No.2402-115-1514).

### Study population

**Fig. 1:**
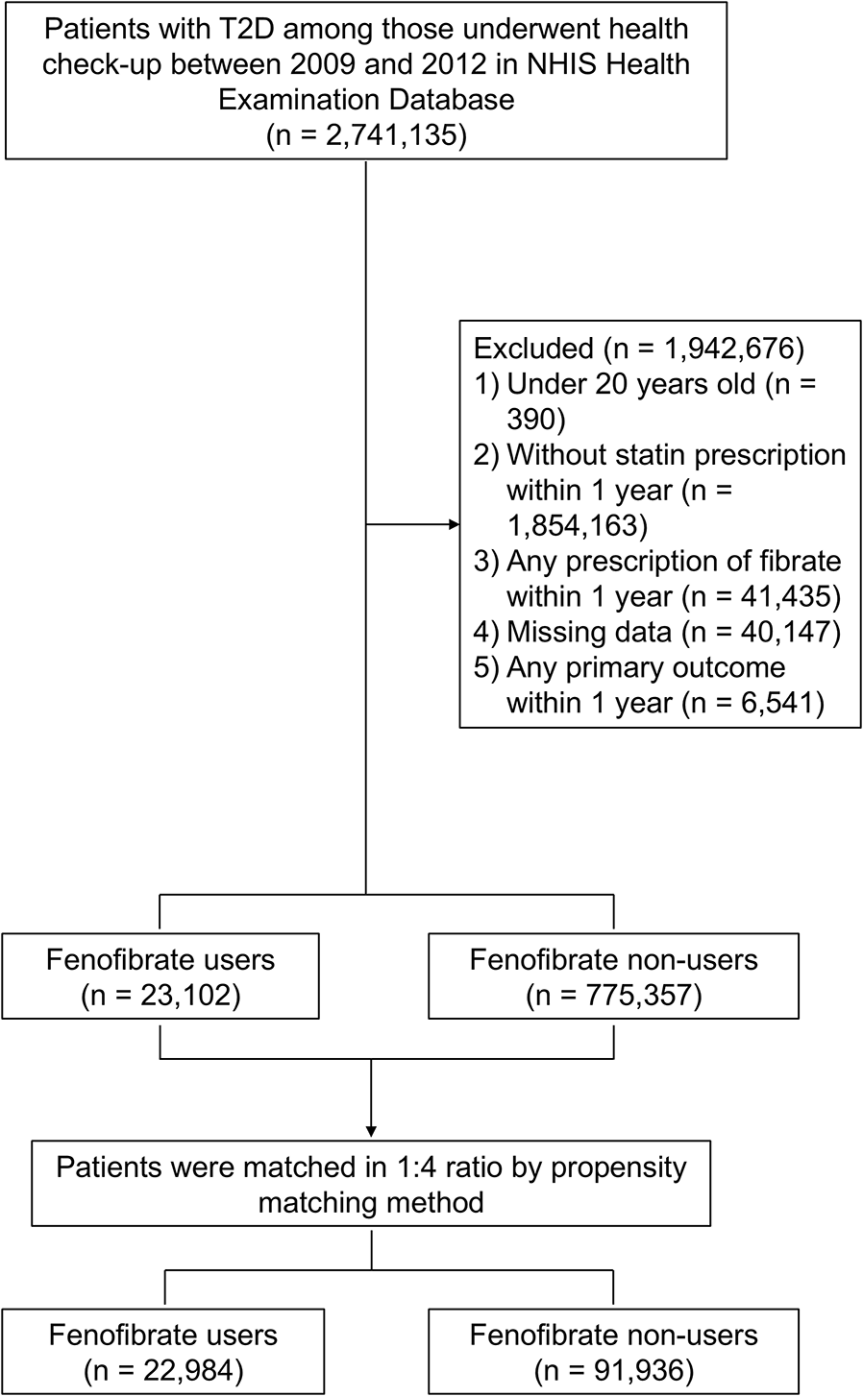
Study flow chart

A total of 2,741,135 patients with T2D was identified from the NHIS Health Examination Database in 2009–2012. Patients with T2D were defined as individuals who had at least one outpatient visit or any hospitalization with a diagnosis using the International Classification of Diseases 10th (ICD-10) revision code for DM (E11–E14) and prescription for antidiabetic agents or fasting plasma glucose (FPG) ≥ 126 mg/dL on health check-up. The following subjects were excluded: 1) < 20 years of age (*n* = 390); 2) without prescription of statin within one year before the index date (*n* = 1,854,163); 3) prescribed any fibrate treatment within one year before the index date (*n* = 41,435); 4) missing information (*n* = 40,147); or 5) PAD or LEA within one year after the index date (*n* = 6,541). The final population enrolled in the study (*n* = 798,459) was divided into two groups based on their history of concomitant statin and fenofibrate prescriptions: fenofibrate users (fenofibrate user group) and non-users (non-user group). The two groups were matched 1:4 using propensity scores. A caliper was set within the first 4 to 8 digits for nearest neighbor matching. In the final analysis, 22,984 and 91,936 patients were included in the fenofibrate user group and non-user group, respectively (Fig. 1).

### Study outcomes

The primary outcomes were defined based on the ICD-10-CM diagnostic codes and admission records. The primary outcomes of the current study were LEA, PAD, or a composite outcome. LEA was defined as hospitalization with surgical codes for amputation (N0562, N0564, N0565, and N0571–N0575) in the lower extremity as the primary diagnosis. PAD was defined as the recording of a related diagnostic code (I702, I708, I709, I739, and I792) and at least one code for an endovascular procedure (M6597, M6605, M6613, and M6620) or surgical procedure (O0163-O0170, O2064, O2065, O2067, and O2068) (Supplementary Table 1). This study compared the occurrence of each clinical outcome between the fenofibrate user group and the non-user group. Follow-up was assessed from the start of the index treatment (starting fenofibrate) until the first occurrence of each outcome, discontinuation of the index treatment for more than four weeks, or the end of the study, whichever came first. Safety-related outcomes included the occurrence of acute kidney injury (N17) and rhabdomyolysis (M62.82) during the observation period and resulting hospitalizations. These outcomes were compared between the two groups.

### Statistical analysis

Continuous variables were described as either mean and standard deviation (SD) or geometric mean with 95% confidence interval (CI) depending on the normality of their distribution. Categorical variables were presented as numbers and percentages. Propensity score (PS) matching was performed to compare the fenofibrate user and non-user group. The PS was determined by calculating the predicted probability of belonging to either the fenofibrate user or non-user group, employing a logistic regression model that included the following covariates: age, sex, body mass index, systolic and diastolic blood pressure, smoking habit (categorized as never smoker, former smoker, and current smoker), alcohol consumption (categorized as a non-drinker, mild drinker, and heavy drinker based on the amount of alcohol consumption per day of 0 g, < 30 g, and ≥ 30 g, respectively), regular exercise (defined as moderate physical activity for ≥ 30 min on ≥ 5 days per week or vigorous physical activity for ≥ 20 min on ≥ 3 days per week), income level (defined as lowest quartile for Q1 and highest quartile for Q4), urban residence, hypertension, chronic kidney disease (CKD, defined as an estimated glomerular filtration rate [eGFR] < 60 mL/min/1.73 m^2^ measured at index year), duration of diabetes (< 5 years or ≥ 5 years), insulin user, oral antidiabetic medication (< 3 classes vs. ≥ 3 classes), fasting blood glucose, total cholesterol, triglyceride, HDL-C, LDL-C, and eGFR. To enhance the robustness of the statistical analysis, each patient in the fenofibrate user group was matched with four patients in the non-user group. The absolute standardized difference (ASD) was used to assess covariate balance, with values of < 0.1 considered indicative of balance.

The incidence rate (IR) for each outcome was calculated by dividing number of events by follow-up time (person-years), expressed in units of 1,000 person-years. Cox proportional hazards regression model was used for analysis of the event occurrence risk. The control group, consisting of patients not treated with fenofibrate, was used as the reference group. The hazard ratio (HR) and corresponding 95% CI were calculated.

Subgroup analyses were performed to assess whether the risk for primary outcomes was consistent for the fenofibrate user group compared with the non-user group in different subgroups: age < 65 years vs. ≥ 65 years); male vs. female; LDL-C < 100 mg/dL vs. ≥ 100 mg/dL; low HDL-C (40 mg/dL and 50 mg/dL for males and females, respectively) and high triglycerides (150 mg/dL) or not; duration of diabetes < 5 years vs. ≥ 5 years; concomitant CKD (-) vs. (+); and multiple antidiabetic combination treatments with < 3 classes vs. ≥ 3 classes. Cox regression analysis was conducted as the primary analysis after adjustment for potential confounders.

Statistical analyses were performed using the SAS software package (SAS version 9.4: SAS Institute, Inc., Cary, NC, USA). All analyses were considered significant with a two-sided p-value < 0.05 and p for interaction < 0.1.

## Results

### Baseline characteristics of fenofibrate users and non-users

The study included 798,459 T2D subjects ≥ 20 years of age identified between 2009 and 2012 from the Korean NHIS database. Finally, 22,984 subjects were defined as fenofibrate users and 91,936 as fenofibrate non-users after PS matching (Fig. 1). The baseline characteristics before PS matching were detailed in Supplementary Table 2. Before PS matching, fenofibrate users were younger than non-users, had a higher proportion of men, were more obese, had a higher proportion of current smokers and heavy alcohol consumers, and higher baseline triglyceride levels (all ASD > 0.1) (Supplementary Table 2. Table 1 summarizes the baseline clinical characteristics of the study population with a comparison of individuals in the fenofibrate user and non-user groups after PS matching. Subsequent to PS matching, the study covariates were well balanced between the two groups (all ASD < 0.1). Serum triglyceride levels were 235.0 mg/dL (95% CI 233.4–236.6) and 234.9 mg/dL (95% CI 234.1–235.7) in the fenofibrate users and non-users, respectively, and were also well balanced for clinical characteristics including sex, age, duration of diabetes, or lifestyle behaviors like smoking, alcohol consumption, and exercise, and other metabolic components.

**Table 1:** Baseline characteristics of study population after matching

Variable	Fenofibrate user (*n* = 22,984)	Fenofibrate non-user (*n* = 91,936)	ASD
Men, n (%)	13,371 (58.2)	53,665 (58.4)	0.004
Age, year	57.5 ± 10.2	57.4 ± 10.7	0.007
BMI, kg/m^2^	26.0 ± 3.2	26.0 ± 3.3	0.002
SBP, mmHg	129.1 ± 15.3	129.2 ± 15.3	0.004
DBP, mmHg	79.3 ± 9.9	79.3 ± 10.1	0.003
Smoking, n (%)			
Current smoker	6,082 (26.5)	24,414 (26.6)	0.002
Former smoker	4,592 (20.0)	18,409 (20.0)	0.001
None	12,310 (53.6)	49,113 (53.4)	0.003
Alcohol consumption, n (%)			
Heavy	2,555 (11.1)	10,195 (11.1)	0.001
Mild	7,285 (31.7)	29,421 (32.0)	0.006
None	13,144 (57.2)	52,320 (56.9)	0.006
Regular exercise, n (%)	4,891 (21.3)	19,543 (21.3)	0.001
Income, n (%)			
Q1	4,890 (21.3)	19,785 (21.5)	0.006
Q2	4,091 (17.8)	16,317 (17.8)	0.001
Q3	5,848 (25.4)	23,286 (25.3)	0.003
Q4	8,155 (35.5)	32,548 (35.4)	0.002
Urban residents	10,477 (45.6)	41,720 (45.4)	0.004
Hypertension, n (%)	16,984 (73.9)	67,972 (73.9)	0.001
CKD, n (%)	3,040 (13.2)	12,074 (13.1)	0.003
Duration of diabetes ≥ 5 years	9,196 (40.0)	36,564 (39.8)	0.005
Insulin user, n (%)	2,567 (11.2)	10,263 (11.2)	0.001
OADs ≥ 3 classes, n (%)	5,363 (23.3)	21,415 (23.3)	0.001
Class of OADs			
Metformin	16,000 (69.6)	61,615 (67.0)	0.056
Sulfonylurea	13,251 (57.7)	52,387 (57.0)	0.014
Meglitinide	619 (2.7)	2,271 (2.5)	0.014
Thiazolidinedione	2,123 (9.2)	9,172 (10.0)	0.025
DPP4 inhibitor	3,453 (15.0)	12,669 (13.8)	0.035
α-glucosidase inhibitor	3,578 (15.6)	14,645 (15.9)	0.010
Fasting glucose, mg/dL	142.5 ± 47.0	142.8 ± 46.5	0.005
Total cholesterol, mg/dL	189.6 ± 46.8	189.9 ± 47.1	0.007
Triglycerides, mg/dL	235.0(233.4-236.6)	234.9(234.1-235.7)	0.001
HDL-C, mg/dL	47.8 ± 17.4	47.8 ± 16.4	0.003
LDL-C, mg/dL	90.0 ± 46.5	90.4 ± 44.4	0.007
eGFR, mL/min/1.73m^2^	84.5 ± 36.5	84.6 ± 41.5	0.003

Data are expressed as mean ± standard deviation (SD) or geometric mean (95% confidence interval) for continuous variables, depending on their normality distribution, and as number (%) for categorical variables. BMI, body mass index; CKD, chronic kidney disease; DBP, diastolic blood pressure; DPP4 inhibitor, dipeptidyl peptidase 4 inhibitor; eGFR, estimated glomerular filtration rate; HDL-C, high-density lipoprotein cholesterol; LDL-C, low-density lipoprotein cholesterol; OADs, oral antidiabetic drugs; SBP, systolic blood pressure

### Clinical outcomes

**Fig. 2:**
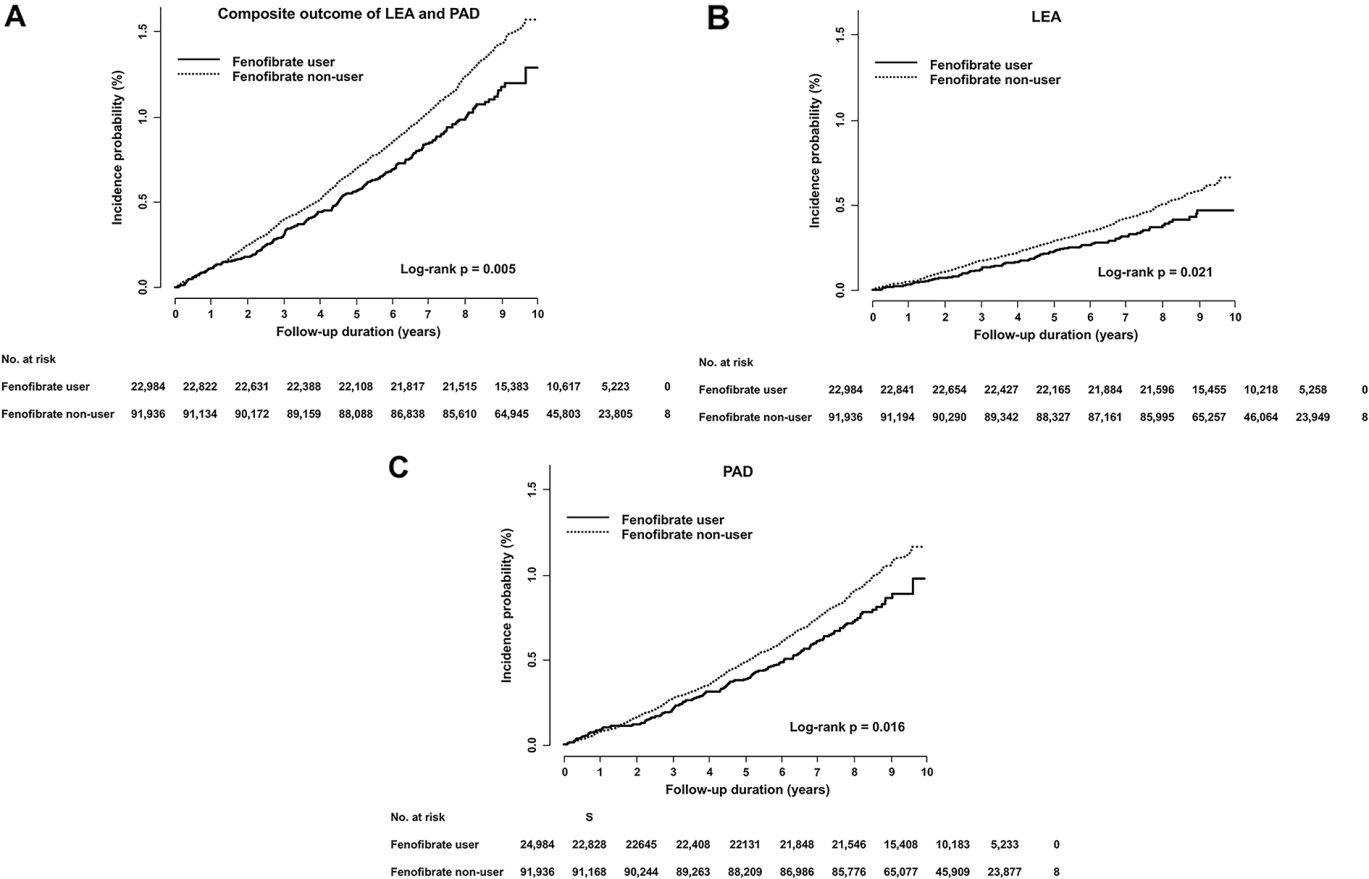
Incidence probabilities of composite and individual outcomes based on fenofibrate user *versus* non-user groups. (A) Incidence probability for the composite outcome of LEA and PAD based on fenofibrate users *versus* non-users (log-rank *p* = 0.005). (B) Incidence probability for LEA based on fenofibrate users *versus* non-users (log-rank *p* = 0.021). (C) Incidence probability for PAD based on fenofibrate users *versus* non-users (log-rank *p* = 0.016). LEA, lower extremity amputation; PAD, peripheral arterial disease

Over a median follow-up of 7.6 years, LEA, PAD, and the composite outcome occurred in 82, 156, and 216 subjects, respectively, in the fenofibrate user group and in 441, 786, and 1,084 subjects, respectively, in the non-user group. Compared with the non-user group, the fenofibrate user group had a significantly lower IR and HR of the composite outcome (1.24 vs. 1.54 per 1,000 person-years; HR 0.81; 95% CI 0.70–0.94; *P* = 0.005, Table 2). In the fenofibrate user group, the IR and HR of LEA was lower than in the non-user group (0.47 vs. 0.62 per 1,000 person-years; HR 0.76; 95% CI 0.60–0.96; *P* = 0.021). In addition, the IR and HR of PAD was significantly lower in the fenofibrate user group than in the non-user group (0.90 vs. 1.11 per 1,000 person-years; HR 0.81; 95% CI 0.68–0.96; *P* = 0.015). Kaplan-Meier analysis showed that the fenofibrate user group showed a significantly lower incidences of LEA, PAD, and the composite outcome compared with the non-user group (all log-rank *P* < 0.05) (Fig. 2). The risk of acute kidney injury, rhabdomyolysis, or hospitalization for these events showed no significant difference between the two groups (Supplementary Table 3).

**Table 2:** Hazard ratios of composite outcome and individual outcomes based on the fenofibrate use status

	Composite outcome (LEA and PAD)			LEA			PAD		
	No. of events	Incidence rate*	HR (95% CI)	No. of events	Incidence rate*	HR (95% CI)	No. of events	Incidence rate*	HR (95% CI)
Fenofibrate users (*n* = 22,984)	216	1.24	0.81 (0.70–0.94)	82	0.47	0.76 (0.60–0.96)	156	0.90	0.81 (0.68–0.96)
Fenofibrate non-users (*n* = 91,936)	1,084	1.54	Reference	441	0.62	Reference	786	1.11	Reference

* Incidence rate per 1000 person-years. HR, hazard ratio; CI, confidence interval; LEA, lower extremity amputation; PAD, peripheral arterial disease

### Subgroup analyses

**Fig. 3:**
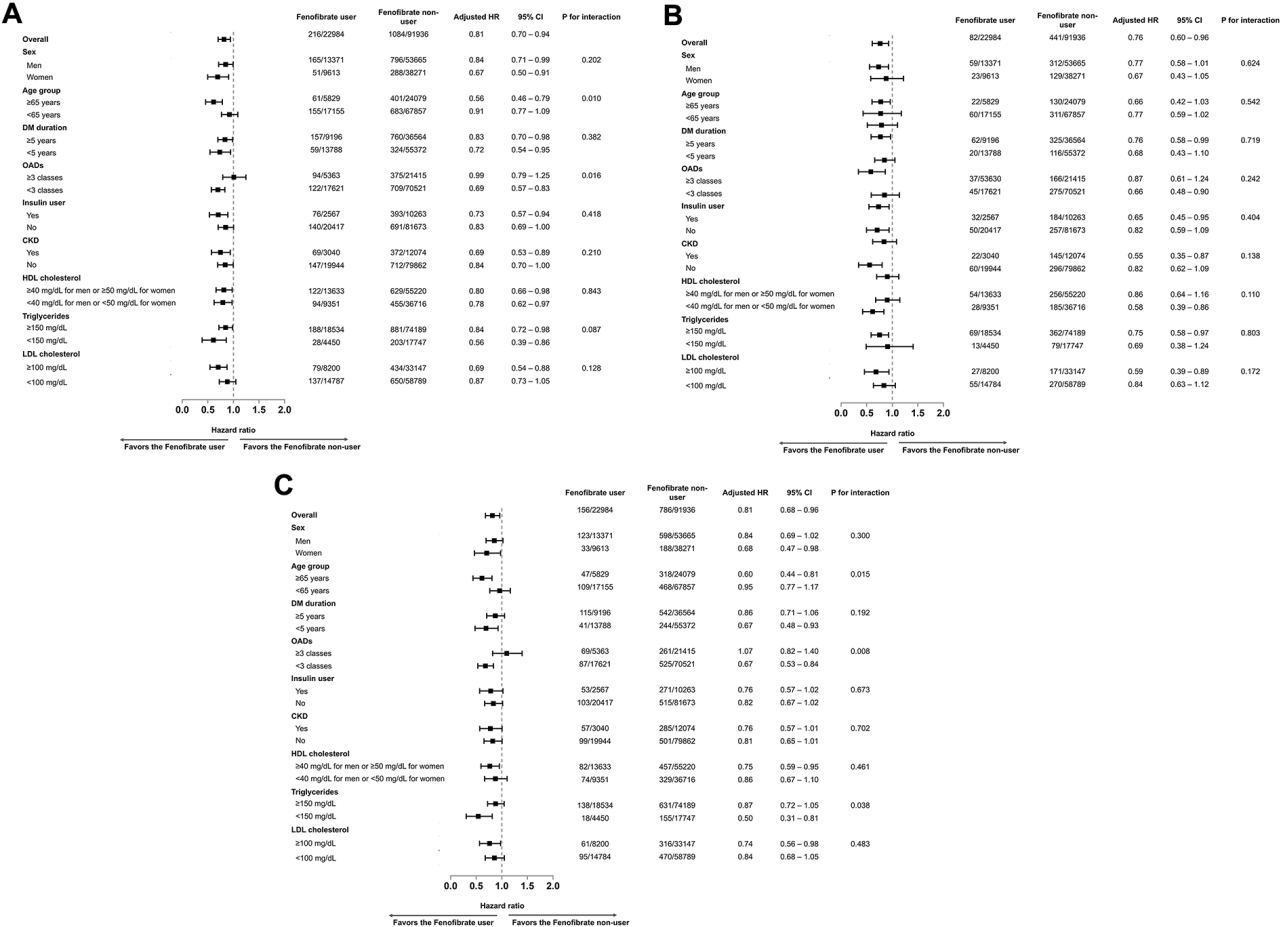
Forest plot of the subgroup analysis showing the HRs for the primary outcome in the pre-specified subgroups: (A) composite outcome, (B) LEA, and (C) PAD. Horizontal bars represent 95% CIs. P-values are for the test of interaction. HRs, hazard ratios; LEA, lower extremity amputation: PAD, peripheral arterial disease; CIs, confidence intervals; CKD, chronic kidney disease; DM, diabetes mellitus; HDL-C, high-density lipoprotein cholesterol; OADs, oral antidiabetic drugs; LDL-C, low-density lipoproteincholesterol

Figure 3 shows the results of subgroup analyses. Significant interactions were observed in the predefined subgroups for relative risk reduction of outcome variables with fenofibrate treatment: age (< 65 years vs. ≥ 65 years), number of oral antidiabetic drug combination therapy classes (< 3 or ≥ 3 classes), and triglyceride level (< 150 mg/dL vs. ≥150 mg/dL) (all *p* < 0.1 for interaction). A significant interaction was found between age and the composite outcome; fenofibrate use was more effective in older patients (≥ 65 years) compared to those under 65 years (adjusted HR [aHR] 0.56; 95% CI 0.46–0.79 vs. aHR 0.91; 95% CI 0.77–1.09 for age ≥ 65 years vs. <65 years, respectively, *p* = 0.010 for interaction). A significant interaction was observed between multiclass antidiabetic combination therapy and the composite outcome, with fenofibrate showing a greater relative risk reduction in the subgroup using < 3 antidiabetic classes compared with the subgroup using ≥ 3 antidiabetic classes. The number of oral antidiabetic agent classes was consistently associated with a lower risk of PAD (aHR 0.67; 95% CI 0.48–0.93 for < 3 classes and aHR 1.07; 95% CI 0.82–1.40; *p* = 0.008 for interaction). The effect of fenofibrate treatment on the composite outcome was significantly higher in the subgroup with lower triglyceride (< 150 mg/dL) than higher triglyceride (≥ 150 mg/dL) (aHR 0.56; 95% CI 0.39–0.86 for lower triglyceride subgroup and aHR 0.84; 95% CI 0.72–0.98 for higher triglyceride subgroup, *p* = 0.087 for interaction). Similar trend was observed in PAD. A significant interaction was observed for PAD, with a marked reduction in the risk in patients with lower triglyceride level (aHR 0.50; 95% CI 0.31–0.81 for triglyceride < 150 mg/dL and aHR 0.87; 95% CI 0.72–1.05 for triglyceride ≥ 150 mg/dL; *p* = 0.038 for interaction). Significant interactions with the outcome variables were not observed in any of the other subgroups based on sex, duration of diabetes, concomitant CKD, HDL-C, LDL-C, or use of insulin.

## Discussion

In this large-scale, population-based nationwide cohort study, the results showed that use of fenofibrate among patients with T2D who had been on statin therapy for > 1 year was associated with significant reductions in the risk of composite outcome by 18.9%, of LEA by 24.2%, and of PAD by 19.1% compared with non-users. Notably, the benefits of fenofibrate were more pronounced in older patients (age ≥ 65 years), those receiving less intensive antidiabetic therapy (< 3 combination therapies), and particularly in individuals with lower triglyceride levels, in which the risk reduction for PAD was most significant.

Current national and international guidelines for the management of diabetes recommend the control of modifiable risk factors to reduce the risk of major adverse cardiovascular events or major adverse limb events, including peripheral revascularization or major amputation, in patients with PAD [9, 10, 22, 23]. In a previous systematic review and meta-analysis, statin use was associated with a significant 30% reduction in major adverse limb events and amputations (pooled HR 0.70; 95% CI 0.61–0.82 and pooled HR 0.65; 95% CI 0.52–0.82, respectively) [24]. Due to the atherogenic dyslipidemia of patients with T2D, fibrates may be a logical therapeutic option for residual risk that is not fully resolved by statins because fibrates have been shown to have beneficial effects on HDL-C, triglycerides, and anti-inflammatory properties [18, 25–28].

Therapeutic options for PAD have been driven mainly by research on atherosclerotic cardiovascular diseases such as coronary heart disease and stroke [23]. Fenofibrate activates peroxisome proliferator-activated receptor-α, which influences the expression of a variety of genes involved in fatty acid oxidation, lipoprotein lipase synthesis, and reduction of apolipoprotein C-III [27]. Consequently, fenofibrate effectively lowers plasma triglycerides and raises HDL-C level. Furthermore, fenofibrates have been shown to exhibit anti-inflammatory and antioxidative effects and to improve endothelial function [29–31]. In several previous randomized controlled trials (RCTs), the efficacy of fenofibrate in reducing cardiovascular events was reported in patients with T2D; however, these studies had relatively small sample sizes (ranging from 164 to 769 patients with T2D) [32–34]. In recent large RCTs, fenofibrate failed to achieve statistical significance in the prevention of cardiovascular events [20, 35]. The results of FIELD did not significantly differ between the fenofibrate and placebo groups in the primary outcome of coronary heart disease events at five years of follow-up [20]. However, the secondary outcomes showed a significant reduction of 11% in total cardiovascular events, 21% in coronary revascularization, and 20% in total revascularization. The FIELD study was conducted with T2D patients who were not on lipid-modifying therapy at baseline. By the end of the study, 33.8% (n = 1,657) and 18.2% (n = 890) of the fenofibrate and placebo groups, respectively, had started using statins. This may explain the lack of statistical significance for the primary outcome of the FIELD study. This study is a retrospective cohort study and differs significantly in methodology and study population from the FIELD study, which was an RCT. Specifically, we examined the effect of fenofibrate on PAD and LEA in patients with T2D who had been taking statins for at least one year. In addition, the fenofibrate group in this study had higher median baseline triglyceride levels (235.0 mg/dL, 95% CI 233.4–236.6) compared with fenofibrate users in the FIELD study (153.1 mg/dL, IQR 118.6–203.5), which may explain the more pronounced effect of fenofibrate in reducing PAD and LEA in the present study. The ACCORD-LIPID trial, which is another representative large scale RCT in which the cardiovascular benefits of fenofibrate were compared in patients with T2D on statin, also failed to demonstrate a significant beneficial effect of fenofibrate over placebo for the composite primary outcome of nonfatal myocardial infarction, nonfatal stroke, or cardiovascular mortality [35]. Although significant interactions were not found between subgroups, the incidence of the primary outcome was lower in the fenofibrate group (12.4%) compared with the placebo group (17.3%) in the subgroup of patients with high triglyceride levels (≥ 204 mg/dL) and low HDL-C levels (≤ 34 mg/dL), indicating that fenofibrate treatment may be beneficial in certain patient populations (p = 0.057 for interaction). In this study, the benefit of fenofibrate on PAD was pronounced in the subgroup with lower triglyceride levels, based on a pre-specified subgroup analysis (p = 0.038 for interaction), supporting the importance of consistently maintaining triglycerides within normal range. In addition, fenofibrate was associated with a significant 25% reduction in the primary outcome in the subgroup taking ≤ 3 oral antidiabetic drugs (OADs; p = 0.011 for interaction). In a 4.9-year extension study consisting of the ACCORD-Lipid survivors (ACCORDION), patients receiving a combination of statin and fenofibrate exhibited a 35% improvement in survival compared with subjects treated with statin alone (aHR 0.65; 95% CI 0.45–0.94; p = 0.02) [36]. Based on subgroup analysis in the present study, this finding indicates a potential ‘legacy effect’ of early fenofibrate intervention, potentially leading to improved outcomes, especially when initiated prior to the progression of diabetes or the requirement for a more complex regimen of OADs for effective glycemic control. Meanwhile, our study showed that the composite outcome of LEA and PAD exhibited a 37.1% reduction in the older patients (age ≥ 65 years), compared to a 9.1% reduction in younger patients (age < 65 years) (*p* = 0.021 for interaction). This provides evidence that proactive lipid lowering therapy with fenofibrate and statins could help prevent major adverse limb events, even in older patients with T2D.

The present study had several limitations. Because this was a population-based cohort study, challenges existed in establishing causal relationship. Despite PS matching being performed to mitigate confounding factors, the possibility of residual unaccounted biases cannot be entirely excluded. However, the findings either align with or serve as robust supplementary evidence to the trends observed in previous comprehensive clinical studies. Such evidence supports the proactive consideration of fenofibrate as a treatment strategy to mitigate major adverse limb events in particular subsets of patients with diabetes who are concurrently undergoing statin therapy. The dataset used in this study primarily captured the sociodemographic characteristics and health behaviors of the South Korean population, limiting the generalizability of the findings to other ethnicities. An analysis of data from the 2013–2015 Korean National Health and Nutrition Examination Survey revealed a high consumption of carbohydrates, contrasted with low intake levels of meat, fish, eggs, legumes, and dairy products [37]. This analysis further identified that males with high carbohydrate intake were inclined toward a 1.35-fold higher odds ratio for developing metabolic syndrome, and females with similar dietary patterns exhibited a 1.38-fold higher odds ratio for reduced HDL-C levels. The results of the present study mirror the prevalent carbohydrate-dominant dietary habits in South Korea, potentially influenced by alcohol consumption habits associated with hypertriglyceridemia. Consequently, this underscores a possibility that the effectiveness of fenofibrate is accentuated in populations characterized by high carbohydrate consumption or in ethnic groups with prevalent high alcohol intake [38].

## Conclusions

The large-scale real-world evidence in the present study indicated that fenofibrate treatment in individuals with T2D was associated with a reduced risk of major adverse limb events, including LEA and PAD, throughout a median observation period of 7.6 years. These findings highlight the valuable role of fenofibrate in reducing the risk of serious vascular complications associated with atherogenic dyslipidemia in patients with T2D.

## Electronic supplementary material

Supplementary Material 1.

## Data Availability

No datasets were generated or analysed during the current study.
