# Supplementary material for: Fenofibrate to prevent amputation and reduce vascular complications in patients with diabetes: FENO-PREVENT
Source: Cardiovasc Diabetol. 2024 Sep 3;23:329. doi: 10.1186/s12933-024-02422-9 (PMC11373174; doi:10.1186/s12933-024-02422-9)
Supplement: Supplementary file 1 — Supplementary Material 1. [file 12933_2024_2422_MOESM1_ESM.docx]

**Supplementary Materials**

**Fenofibrate to Prevent Amputation and Reduce Vascular Complications in Patients with Diabetes: FENO-PREVENT**

Eu Jeong Ku^1,2^, Bongseong Kim^3^, Kyungdo Han^3^, Seung-Hwan Lee^4,5^, Hyuk-Sang Kwon^6^

^1^ Department of Internal Medicine, Seoul National University Hospital Healthcare System Gangnam Center, Seoul, South Korea

^2^ Department of Internal Medicine, Seoul National University College of Medicine, Seoul, South Korea

^3^ Department of Statistics and Actuarial Science, Soongsil University, Seoul, South Korea

^4^ Division of Endocrinology and Metabolism, Department of Internal Medicine, Seoul St. Mary's Hospital, College of Medicine, The Catholic University of Korea, Seoul, South Korea

^5^ Department of Medical Informatics, College of Medicine, The Catholic University of Korea, Seoul, South Korea

^6^ Division of Endocrinology and Metabolism, Department of Internal Medicine, Yeouido St Mary's Hospital, The Catholic University of Korea, Seoul, South Korea

**Contents**

**Table S1**. Surgery and procedure codes and descriptions.

**Table S2.** Baseline characteristics of study population before matching

**Table S3.** Hazard ratios of acute renal failure, rhabdomyolysis and hospitalization for these events based on the fenofibrate use status.

**Table S1**. Surgery and procedure codes and descriptions

| Code | Description |
| --- | --- |
| N0562 | Disarticulation of extremities at hip |
| N0564 | Disarticulation of extremities at knee |
| N0565 | Disarticulation of extremities at elbow, wrist, or ankle |
| N0571 | Amputation of extremities at pelvis |
| N0572 | Amputation of extremities at thigh |
| N0573 | Amputation of extremities at upper arm, forearm, or lower leg |
| N0574 | Amputation of extremities at hand or foot |
| N0575 | Amputation of extremities at finger, or toe |
| I702 | Atherosclerosis of native arteries of the extremities |
| I708 | Atherosclerosis of other arteries |
| I709 | Other and unspecified atherosclerosis, unspecified atherosclerosis, generalized atherosclerosis, chronic total occlusion of artery of the extremities |
| I739 | Peripheral vascular disease, unspecified |
| I792 | Other atherosclerosis of other type of bypass graft(s) of the extremities |
| M6597 | Percutaneous transluminal angioplasty at others |
| M6605 | Percutaneous intravascular installation of metallic stent at others |
| M6613 | Percutaneous intravascular installation of stent-graft at others |
| M6620 | Percutaneous intravascular atherectomy |
| O0163 | Vascular bypass operation (artery) of femoral-popliteal (above knee joint) with autologous vessel |
| O0164 | Vascular bypass operation (artery) of femoral-popliteal (above knee joint) with artificial vessel |
| O0165 | Vascular bypass operation (artery) of femoral-popliteal (below knee joint) with autologous vessel |
| O0166 | Vascular bypass operation (artery) of femoral-popliteal (below knee joint) with artificial vessel |
| O0167 | Vascular bypass operation (artery) of femoral-tibia, fibula with autologous vessel |
| O0168 | Vascular bypass operation (artery) of femoral-tibia, fibula with artificial vessel |
| O0169 | Vascular bypass operation (artery) of popliteal-tibia, fibula with autologous vessel |
| O0170 | Vascular bypass operation (artery) of popliteal-tibia, fibula with artificial vessel |
| O2064 | Transluminal atherectomy at abdominal artery or iliac artery |
| O2065 | Transluminal atherectomy at others |
| O2067 | Transluminal atherectomy at abdominal artery or iliac artery and patch repair |
| O2068 | Transluminal atherectomy at others and patch repair |

**Table S2.** Baseline characteristics of study population before matching

| Variable | Fenofibrate user  (n=23,102) | Fenofibrate non-user  (n=775,357) | ASD |
| --- | --- | --- | --- |
| Men, n (%) | 13,479 (58.4) | 382,104 (49.3) | 0.183 |
| Age, year | 57.4 ± 10.3 | 60.9 ± 10.3 | 0.338 |
| BMI, kg/m^2^ | 26.0 ± 3.2 | 25.4 ± 3.3 | 0.176 |
| SBP, mmHg | 129.1 ± 15.3 | 128.7 ± 15.5 | 0.026 |
| DBP, mmHg | 79.3 ± 9.9 | 78.0 ± 10.0 | 0.131 |
| Smoking, n (%) |  |  |  |
| Current smoker | 6,154 (26.6) | 139,720 (18.0) | 0.208 |
| Former smoker | 4,615 (20.0) | 144,433 (18.6) | 0.034 |
| None | 12,333 (53.4) | 491,204 (63.4) | 0.203 |
| Alcohol consumption, n (%) |  |  |  |
| Heavy | 2,604 (11.3) | 47,545 (6.13) | 0.183 |
| Mild | 7,339 (31.8) | 199,099 (25.7) | 0.135 |
| None | 13,159 (57.0) | 528,713 (68.2) | 0.234 |
| Regular exercise, n (%) | 4,925 (21.3) | 173,372 (22.4) | 0.025 |
| Income, n (%) |  |  |  |
| Q1 | 4,914 (21.3) | 160,289 (20.7) | 0.015 |
| Q2 | 4,108 (17.8) | 132,632 (17.1) | 0.018 |
| Q3 | 5,875 (25.4) | 189,326 (24.4) | 0.023 |
| Q4 | 8,205 (35.5) | 293,110 (37.8) | 0.047 |
| Urban residents | 10,542 (45.6) | 357,286 (46.1) | 0.009 |
| Hypertension, n (%) | 17,070 (73.9) | 575,661 (74.2) | 0.008 |
| CKD, n (%) | 3,044 (13.2) | 116,249 (15.0) | 0.052 |
| Duration of diabetes ≥5 years | 9,227 (39.9) | 345,699 (44.6) | 0.094 |
| Insulin user, n (%) | 2,580 (11.2) | 92,769 (12.0) | 0.025 |
| OADs ≥3 classes, n (%) | 5,406 (23.4) | 173,231 (22.3) | 0.025 |
| Class of OADs |  |  |  |
| Metformin | 16,087 (69.6) | 529,676 (68.3) | 0.029 |
| Sulfonylurea | 13,325 (57.7) | 435,039 (56.1) | 0.032 |
| Meglitinide | 627 (2.7) | 20,274 (2.6) | 0.006 |
| Thiazolidinedione | 2,135 (9.2) | 84,027 (10.8) | 0.053 |
| DPP4 inhibitor | 3,477 (15.1) | 110,582 (14.3) | 0.022 |
| α-glucosidase inhibitor | 3,598 (15.6) | 120,953 (15.6) | 0.001 |
| Fasting glucose, mg/dL | 142.5 ± 47.0 | 137.6 ± 44.9 | 0.106 |
| Total cholesterol, mg/dL | 189.7 ± 46.7 | 177.5 ± 43.9 | 0.268 |
| Triglycerides, mg/dL | 236.4 (234.8-238.1) | 136.7 (136.6-136.9) | 1.031 |
| HDL cholesterol, mg/dL | 47.7 ± 17.4 | 51.9 ± 21.4 | 0.214 |
| LDL cholesterol, mg/dL | 89.8 ± 46.6 | 95.1 ± 40.7 | 0.121 |
| eGFR, mL/min/1.73m^2^ | 84.6 ± 37.2 | 82.6 ± 35.9 | 0.054 |

Data are expressed as mean ± standard deviation (SD) or geometric mean with 95% confidence interval for continuous variables, depending on their normality distribution, and as number (%) for categorical variables. BMI, body mass index; CKD, chronic kidney disease; DBP, diastolic blood pressure; DPP4 inhibitor, dipeptidyl peptidase 4 inhibitor; eGFR, estimated glomerular filtration rate; HDL cholesterol, high-density lipoprotein cholesterol; LDL, low-density lipoprotein cholesterol; OADs, oral antidiabetic drugs; SBP, systolic blood pressure.

**Table S3.** Hazard ratios of acute renal failure, rhabdomyolysis and hospitalization for these events based on the fenofibrate use status.

|  | No. of events | Incidence rate (1000 person-year) | HR (95% CI) |
| --- | --- | --- | --- |
| Acute renal failure |  |  |  |
| Fenofibrate users (n=22,984) | 1,563 | 9.20 | 1.06 (0.99 – 1.12) |
| Fenofibrate non-users (n=91,936) | 6,039 | 8.75 | Reference |
| Hospitalization for acute renal failure |  |  |  |
| Fenofibrate users (n=22,984) | 1,048 | 6.10 | 1.04 (0.97 – 1.11) |
| Fenofibrate non-users (n=91,936) | 4,120 | 5.90 | Reference |
| Rhabdomyolysis |  |  |  |
| Fenofibrate users (n=22,984) | 7 | 0.04 | 0.79 (0.35 – 1.77) |
| Fenofibrate non-users (n=91,936) | 36 | 0.05 | Reference |
| Hospitalization for rhabdomyolysis |  |  |  |
| Fenofibrate users (n=22,984) | 0 | 0 | NA |
| Fenofibrate non-users (n=91,936) | 3 | <0.01 | Reference |

HR, hazard ratio; CI, confidence interval; NA, not applicable.
